# Simultaneous production of detergent stable keratinolytic protease, amylase and biosurfactant by *Bacillus subtilis* PF1 using agro industrial waste

**DOI:** 10.1016/j.btre.2016.03.007

**Published:** 2016-04-13

**Authors:** Khushboo Bhange, Venkatesh Chaturvedi, Renu Bhatt

**Affiliations:** a Department of Biotechnology, Guru Ghasidas Vishwavidyalaya (A Central University), Bilaspur 495009, Chhattisgarh, India; b School of Biotechnology, Banaras Hindu University, Varanasi, U.P., India

**Keywords:** Protease, Amylase, Biosurfactant, *Bacillus subtilis* PF1

## Abstract

The present study is an attempt to optimize simultaneous production of keratinolytic protease, amylase and biosurfactant from feather meal, potato peel and rape seed cake in a single media by response surface methodology to evaluate their biochemical properties for detergent additive. The optimization was carried out using 20 run, 3 factor and 5-level of central composite design on design expert software which resulted in a 1.2, 0.84 and 2.28 fold increase in protease, amylase and biosurfactant production. The proteolytic activity was found to be optimum at pH 9.0 and 60 °C while optimum amylolytic activity was recorded at pH 6.0 and 70 °C respectively. Both enzymes were found to be stable in the presence of organic solvents, ionic and commercial detergent and oxidizing agents. The biosurfactant was extracted with chloroform and was found to be stable at varying pH and temperature; however a reduction in the activity was observed at temperature higher than 70 °C. The isolated enzymes and biosurfactants may find applications in the effective removal of stains.

## Highlights

• Keratinolytic protease, amylase and Biosurfactant was produced in a single medium.
• Medium composition was optimized statistically in Design Expert software.
• Optimization resulted in a 1.2, 0.84 and 2.28% increase in keratinase, amylase and biosurfactant production.
• The isolated enzymes and biosurfactants may find applications in the effective removal of stains.

## Graphical abstract

**Figure:**
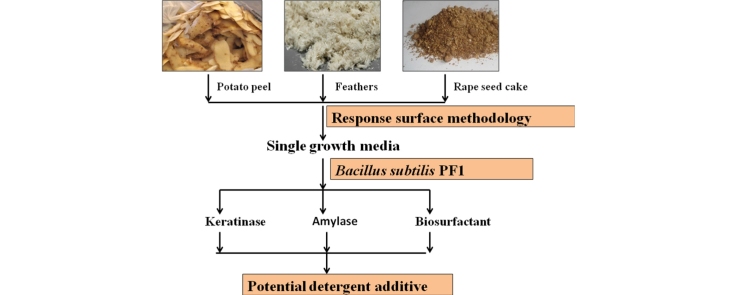

## 1 Introduction

Enzymes are considered as ‘Green chemicals’ and their role in detergent industry is well established [1]. Their use in detergent formulations not only enhances the efficiency to remove stains but also make the detergents environmentally safe [2]. Two important enzymes alkaline proteases and amylases are widely employed in detergent formulations. Keratinases are mostly serine or metallo alkaline proteases capable of degrading keratin protein, a group of fibrous, recalcitrant and abundant structural proteins that constitutes the major component of structure such as feathers, nail, horns and hooves [3]. Keratinases exhibit several important biotechnological applications such as dehairing agent in leather industry, slow release nitrogen fertilizers, cosmetics and detergent industry [4], [5], [6]. Amylase is another most important industrial enzyme which catalyses the breakdown of starch or glycogen into oligosaccharide differing in size [7]. Amylases are indispensable in bread making, brewing, textile, paper, and pharmaceutical and detergent industries [8]. Various industrial processes involved the combination of alkaline proteases and amylases. Beside detergent industries alkaline proteases and amylases are also used together in food industry, pharmaceutical industries [2]. Although several microorganisms are known to produce alkaline proteases and amylases, *Bacillus* strains are often preferred due to their exceptional ability to secrete large amounts of highly active enzymes [9].

Along with the enzymes, role of biosurfactants in detergency have also gained impetus in recent years. Biosurfactants are produced from microbial systems and are characterized by amphiphilic structure [10]. Nowadays, they are widely employed in oil recovery, pharmaceutical industries and metal sequestering [11], [12]. They are known for conferring excellent detergent, emulsifying, foaming and dispersing traits [13]. Due to the increasing public concern about the environmental hazards of synthetic surfactants, the use of ecofriendly surfactants from natural sources in detergent formulations is under consideration [10].

The high cost of substrates and medium used for the production of enzymes is responsible for increased market cost of enzymes [14]. On the other side the valuable agro industrial wastes such as wheat bran, potato peel, soy meal, and chicken feathers remain unutilized and are discarded. The increasing market demand of enzymes necessitates the use of economical and easily available substrates for enzyme production. Most of the studies are focused on the production of single enzyme using these agro industrial wastes. The use of low cost substrates to produce multiple enzymes together with biosurfactant in a single culture media would be time saving, economical and eco friendly.

Response surface methodology (RSM) is a statistical approach of media formulation which provides useful information about interacting factors in limited numbers of experiments [1]. Central composite design (CCD) is a potential method of RSM used to investigate the optimum concentration of media component. Therefore, in the present study concomitant production of keratinolytic protease, amylase and biosurfactant from *Bacillus subtilis* PF1 was optimized by RSM approach. The use of enzymes in detergent formulations offer several particularly hard challenges particularly their compatibility with detergent ingredients, appropriate activity at relevant washing temperature and pH, stain removal potential, stability and half life [1]. Thus, the study was further extended to evaluate the biochemical characteristics of keratinolytic protease, amylase and biosurfactant and finally washing performance of these three factors were investigated.

## 2 Experimental

### 2.1 Materials

Chicken feathers were procured from regional poultry farm of Bilaspur, Chhattisgarh, India. They were washed thoroughly with detergent followed by running tap water for 2–3 times to remove dust and blood stains and, were dried in hot air oven for 24 h at 60 °C. The rape seed cake was purchased from regional shop and potato peel was obtained from kitchen waste. The chemicals and organic salts used in the present study were of pure grade and purchased from Himedia Pvt. Ltd, Mumbai, India.

### 2.2 Microorganism, initial media and culture conditions

*Bacillus subtilis* PF1 (accession no. KM062030) a keratinolytic strain was isolated from the soil of regular feather disposal site of Bilaspur, Chhattisgarh, India. The strain was grown on skimmed milk agar plate (Hi-media, India) by incubating at 32 °C for 24 h to determine proteolytic activity. Similarly, amylase activity was determined by culturing the bacterial cells into starch agar media. Stock culture was maintained in 30% glycerol and stored at −20 °C.

Minimal salt medium (FMB1) used for culturing of bacterial cells which consisted of following components (g/l): K_2_HPO_4_, 0.3; KH_2_PO_4_, 0.4; NaCl, 0.5 and feather 10, potato peel 10, and rape seed cake 5, pH 7.2. Peptone water [(g/l) peptone, 10; and NaCl, 5; pH 7.2] was used for inoculum preparation. All the media described here were autoclaved at 121 °C and 15 psi for 15 min. Culture conditions were maintained at 37 °C and 150 rpm. Strain PF1 was inoculated in 100 ml Erlenmeyer flask containing 50 ml peptone water and incubated at 37 °C and 150 rpm in an orbital shaker (Remi, C-24 BL, India). After 16 h of growth, the culture was centrifuged at 8000 rpm for 10 min in a cooling centrifuge (Remi, C-24 BL, India). The cell pellet was washed twice and suspended in 5 ml minimal media; 1% (v/v) (approximately 10^8^ bacterial cells) of the suspension was then transferred to 100 ml of fresh media and incubated at the same conditions for five days. Aliquots of 2 ml were retrieved from each of triplicate cultures after every 24 h to evaluate the soluble protein production and enzyme activity [15]. All the assays were performed in triplicate for each parameter.

### 2.3 Estimation of soluble protein

Protein concentration was determined by Bradford method [16] using bovine serum albumin (BSA) as standard. The absorbance was determined spectrophotometrically at 590 nm using, UV–vis spectrophotometer (UV-1800, Shimadzu).

### 2.4 Estimation of protease activity

Protease assay was performed according to the method of Anbu et al. [17] with minor modifications. Briefly, 20 mg of casein was suspended in 3.8 ml of 100 mM phosphate buffer (pH 7.0) followed by addition of 200 μl of culture supernatant to it. The preparation was incubated at 50 °C for 1 h. The reaction was stopped by transferring the tubes to ice chilled water. After centrifugation at 10,000 rpm for 5 min, the absorbance of supernatant was measured at 280 nm. Control samples were prepared in a similar manner except that the enzyme was replaced by same volume of 100 mM phosphate buffer (pH 7.0). One unit of enzyme activity was considered as amount of enzyme required to release 1 μmol/min of tyrosine under the conditions mentioned above.

### 2.5 Estimation of amylase activity

Amylase activity was determined by measuring the release of reducing sugar during enzymatic degradation of starch [2]. The reaction mixture contained 500 μl of crude enzyme and 500 μl 1% starch (Himedia, India) as substrate in 100 mM phosphate buffer (pH 7.0). The reaction mixture was incubated at 30 °C for 15 min. The amount of released reducing sugar was determined by dinitrosalisylic acid (DNS) method [18]. One unit of amylase activity was defined as the amount of enzyme that released 1 μmol of reducing sugar per minute. Maltose was used as standard.

### 2.6 Estimation of emulsification index

The biosurfactant emulsification index was determined by a modified method of Ramnani et al. [13]. Water, xylene and culture filtrate were mixed in the ratio of 20:30:10 in a measuring test tube. The height of the solvent layer in the test tube was measured and the mixture was vortexed to create an emulsion. The height of the emulsion layer was measured after 24 h and emulsification index (EI) was calculated using the equation EI (%) = [(height of emulsion layer)/(height of oil + emulsion layer)] × 100. The results were compared with a positive control (water:xylene:Tween-80 = 25:30:5) and negative control (water:xylene = 30:30).

### 2.7 Statistical optimization of protease, amylase and surfactant production using response surface methodology

The optimum concentration and interaction of medium component, namely feather meal, potato peel and rape seed cake was studied by central composite design (CCD) shown in Table 1. Each factor was studied at five different levels −α, −1, 0, +1 and +α. A set of 20 experiments was generated by design expert software. Experiments were run in triplicates and the average value of enzyme activity and emulsification index were taken as dependent variable or response. Twenty sets were prepared and incubated at 37 °C and 150 rpm for 48 h.

### 2.8 Statistical analysis and modelling

The data obtained from CCD were subjected to analysis of variance (ANOVA) and multiple regression analysis was performed by fitting first order polynomial equation. This resulted in an empirical model that related the response measured in the independent variable to the experiment. The model equation was:

$$\text{Y}={\text{B}}_{0}{\text{+B}}_{1}{\text{X}}_{1}{\text{+B}}_{2}{\text{X}}_{2}{\text{+B}}_{3}{\text{X}}_{3}{\text{+B}}_{4}{\text{X}}_{1}{\text{X}}_{2}{\text{+B}}_{5}{\text{X}}_{1}{\text{X}}_{3}{\text{+B}}_{6}{\text{X}}_{2}{\text{X}}_{3}{\text{+B}}_{7}{\text{X}}_{1}^{2}{\text{+B}}_{8}{\text{X}}_{2}^{2}{\text{+B}}_{9}{\text{X}}_{3}^{2}$$

where B_0_–B_9_ are the regression coefficients of the respective variables and interaction terms computed from the observed experimental values of measured response. X_1_–X_3_ are the codes for independent variables. The independent variables were feather meal (g/l) (X_1_), potato peels (g/l) (X_2_) and rape seed cake (g/l) (X_3_). The measured responses Y_1_ = protease activity (U/ml), Y_2_ = amylase activity (U/ml) and Y_3_ = biosurfactant production (emulsification index) with constrains applied are described in Table 1. The responses of variables were analysed by three dimensional plots.

### 2.9 Verification of experimental model

To verify the optimized medium, a time course study to evaluate soluble protein production, enzyme activity and emulsification index by the strain was performed. Aliquots of 2 ml were retrieved from each of the triplicate cultures at an interval of every 24 h to evaluate soluble protein production, enzyme activity and emulsification index. All the assays were performed in triplicate for each parameter.

### 2.10 Partial purification of amylase and protease

After validation of RSM, the optimized protease, amylase and biosurfactant production medium (FMB 2) was prepared which composed of feather meal, 12.5 g/l; potato peel, 12.5 g/l; rape seed cake 6 g/l. Five percent inoculum from overnight grown culture of strain PF1was added in freshly prepared FMB2 medium. One litre of the cell free supernatant was collected from the 72 h culture by centrifugation at 10,000 rpm for 10 min at 4 °C and was subjected to ammonium sulphate precipitation. Ammonium sulphate was added to the supernatant to bring the saturation up to 45%. The precipitate was collected by centrifugation at 12,000*g*, 4 °C for 30 min. The precipitate containing enzyme was recovered by resuspending the pellet in 20 mM phosphate buffer (pH 7.0) and the suspension was dialysed thoroughly against the same buffer for desalting.

### 2.11 SDS-PAGE and zymography

The molecular weight of the partially purified protease and amylase was determined using 12% SDS-PAGE. Gels were run in a mini-protean 3Cell apparatus (Bio-Rad Laboratories Inc., Hercules, USA). Protein bands were detected after staining the gel with silver nitrate. The relative molecular mass of the protease and amylase was determined by comparing with known standard molecular weight markers (97.4–29.0 kDa). For zymography, partially purified protease was loaded onto the native 12% polyacrylamide gel and electrophoresis was conducted at room temperature under non denaturing conditions. The gel was incubated for 12 h in 100 mM Tris HCl buffer (pH 8.0) containing 1% (w/v) casein and then stained with 0.05% (w/v) coomassie brilliant blue dye for 2 h. The activity band was observed after destaining as a clear colourless area in the gel against dark background. Zymography of amylase was performed according to the method of Sahnoun et al. [8]. Briefly, the native gel was placed onto the agarose gel containing 1% starch and incubated at 30 °C for 30 min. After incubation the gel was washed with distilled water and treated with iodine reagent (2% iodine in 0.2% potassium iodine), and amylase activities were visualized as transparent bands on a dark blue background.

### 2.12 Biochemical characterization of protease and amylase

#### 2.12.1 Effect of pH, temperature

To determine the optimum pH, enzyme activity was assayed using different buffers with wide range of pH (4.0–11.0): acetate buffer (100 mM pH 4.0 and 5.0), phosphate buffer (100 mM, pH 6.0–8.0) and glycine-NaOH buffer (100 mM, pH 9.0 and 11.0). The pH stability of the enzyme was determined by the enzyme in different buffers at pH 6–11 for 3 h at standard assay conditions. The optimum temperature for partially purified protease and amylase was determined at the different temperature ranging from 30 to 80 °C. The temperature stability of enzyme was determined by pre incubating the protease and amylase for 3 h at 30–80 °C. The activity of the protease and amylase was then measured under standard assay condition. The residual activity was estimated by taking highest activity as 100%.

#### 2.12.2 Effect of organic solvents, and surfactants

The effect of metal ions on protease and amylase activity was determined by using different salts (CaCl_2_, NaCl, ZnCl_2_, CuSO_4_, MnSO_4_, MgSO_4_) at final concentration of 2 mM. The effect of detergent (SDS, Triton X100), solvent (DMSO), reducing agent (β-mercaptoethanol), oxidizing agent (H_2_O_2_), enzyme inhibitors (EDTA, PMSF, 1,10, phenanthroline) on the enzyme activity was studied by measuring the enzyme activity in the presence of respective chemicals at different concentrations (shown in Table 3) at standard assay conditions. The reaction mixture devoid of any chemical or metal ion was considered as control. The non-treated enzyme containing 1 mM EDTA was considered as 100% for determination of enzyme activity in presence of metal ions.

### 2.13 Compatibility of amylase and protease with commercial detergents

The compatibility of protease and amylase was examined against various commercial laundry detergents available in local market namely, Nirma^®^ (Nirma Ltd., India), Rin^®^ (Hindustan Unilever Ltd., India), Wheel^®^ (Hindustan Unilever Ltd., India), Ghadi^®^ (Rohit Surfactant Pvt. Ltd., India), Surf excel^®^ (Hindustan Unilever Ltd., India), Ariel^®^ (Procter & Gamble, India), Tide^®^ (Procter & Gamble, India). Commercial detergents were diluted in tap water to achieve final concentration of 7 mg/ml (6). The endogenous enzymes present in detergents were inactivated by heating the diluted detergent for 1 h at 70 °C prior to addition of enzyme preparation. The partially purified protease and amylase were incubated with deactivated detergent solution for 1 h at 50 °C and then the remaining activities were determined separately for protease and amylase under standard assay conditions.

### 2.14 Extraction of biosurfactant

The surfactant produced in the optimized media was extracted with equal amount of ethyl acetate. The ethyl acetate was evaporated and the crude extract was dissolved in chloroform [10].

### 2.15 Effect of pH and temperature on emulsification index of biosurfactant

Aqueous solution of extracted biosurfactant was prepared at a concentration of 0.05%. The effect of temperature was studied by incubating the biosurfactant solution at 50–80 °C for 2 h followed by measuring the emulsification index of the solution. The pH stability was studied over a pH range of 6.0–11.0. The residual activity was estimated by taking highest activity as 100%.

### 2.16 Washing performance of protease, amylase and biosurfactants

Application of protease, amylase and biosurfactant mixture as a detergent additive was studied on white cotton cloth pieces (4 × 4 cm) stained with human blood, chocolate and beet root juice. The stained cloth pieces were taken in separate flasks. The following sets were prepared:

1. Flask with distilled water (100 ml) + stained cloth (cloth stained with blood).
2. Flask with distilled water (100 ml) + 1 ml SDS (7 mg/ml) + stained cloth (cloth stained with blood).
3. Flask with distilled water (100 ml) + 1 ml SDS (7 mg/ml) + 2 ml enzyme solution + stained cloth (cloth stained with blood).

The above flasks were incubated at 50 °C for 15 min. After incubation, cloth pieces were taken out, rinsed with water and dried. Visual examination of various pieces exhibited the effect of enzyme in removal of stains. Untreated cloth pieces stained with blood were taken as control [19].

## 3 Results

### 3.1 Production of protease, amylase and biosurfactant in initial culture medium

The culture was grown for evaluation of protease, amylase and biosurfactant production in initial culture medium containing (g/L): K_2_HPO_4_, 0.3; KH_2_PO_4_, 0.4; NaCl, 0.5 and feather 10, potato peel 10, and rape seed cake 5, pH 7.2. Maximum protease activity was obtained 21.9 ± 1.9 U/ml 72 h of incubation. Maximum soluble protein (59.31 ± 2.01 μg/ml) and maximum amylase production (16.39 ± 4.95 μg/ml) was obtained at 96 h of incubation. Biosurfactant production was found to highest 25.93% at 120 h of incubation (data unpublished).

### 3.2 RSM approach for optimization of medium

**Fig. 1:**
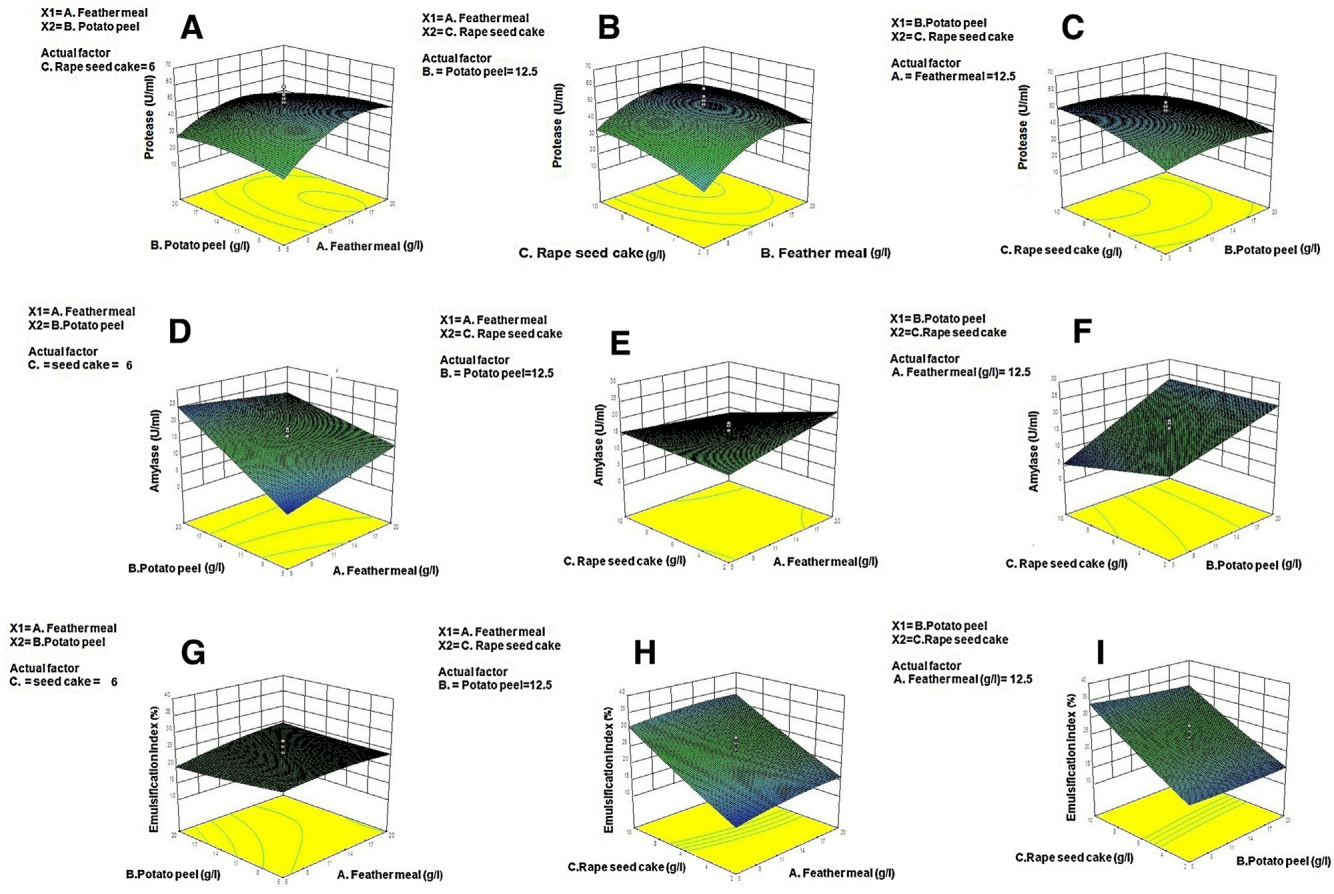
3D response surface plot for effect of feather meal concentration, potato peel and rape seed cake and their interaction terms on protease (A–C), amylase (D–F) and biosurfactant production (G–I).

Using three independent variables i.e. X_1_ = feather meal concentration, X_2_ = potato peel concentration and X_3_ = rape seed cake concentration, 20 set of experiments were performed applying central composite design (CCD) to optimize production of protease, amylase and biosurfactants. The model predicted the mean protease production of 48.85 U/ml, mean amylase production 16.56 U/ml and emulsification index of biosurfactant production 23.69 in medium containing (g/l): feather meal, 12.5; potato peel, 12.5; and rape seed cake 6. The three dimensional graphs from which protease, amylase and biosurfactant production were evaluated was plotted against two variables at a time by keeping the value of one variable fixed at middle level (Fig. 1).

### 3.3 Protease production

As shown in Table 1, the values of protease production were in range of 11.1–54.75 U/ml (Table 1). The experimental data was used to calculate the regression coefficients of the quadratic polynomial equation and the significance of each regression coefficient was statistically evaluated by ANOVA Table 2. The *P*-value for the model (0.02) and for “Lack of fit” (0.4) also suggested that the obtained experimental data was good fit with the model. The response of protease production (Y_1_) regression by strain *Bacillus subtilis* PF1 can be expressed in terms of following regression equation:

$${\text{Y}}_{1}=48{\text{.48+6.76X}}_{1}-{\text{2.19X}}_{2}{\text{+4.74X}}_{3}-{\text{3.20X}}_{1}{\text{X}}_{2}-{\text{1.79X}}_{1}{\text{X}}_{3}-{\text{2.65X}}_{2}{\text{X}}_{3}-\text{10.12}{\text{X}}_{1}^{2}-\text{3.51}{\text{X}}_{2}^{2}-\text{3.54}{\text{X}}_{3}^{2}$$

The equation clearly explained that the presence of variable X_1_ and X_3_ i.e. feather meal and rape seed cake positively influenced the production of protease. Whereas, the presence of potato peel (X_2_) negatively affects the production. The interacting effect of X_1_X_2_, X_1_X_3_ and X_2_X_3_ and also shows negative effect on protease production. The value of multiple regression coefficient (R^2^ = 0.8) indicated agreeable correlation between predicted and observed value. The reliability of the statistical model was confirmed by low value of coefficient of variation (24.48%). The 3D response surface plot has been shown Fig. 1A–C.

### 3.4 Amylase production

The amylase production varied from 4.61 to 27.9 U/ml (Table 1) for various combinations. The *P*-value for the model (<0.0001) suggested the significance of the model and the value of “Lack of fit” (0.3) indicated that the obtained experimental data was good fit with the model (Table 2). The effect of 3 variables on amylase production can be explained by following equation:

$${\text{Y}}_{2}={\text{16.56+1.21X}}_{1}{\text{+6.90X}}_{2}-{\text{1.81X}}_{3}-{\text{2.98X}}_{1}{\text{X}}_{2}-{\text{2.67X}}_{1}{\text{X}}_{3}{\text{+1.97X}}_{2}{\text{X}}_{3}$$

The value of correlation coefficient (R^2^) for amylase production was found to be 0.88, representing a good fit. The variable X_2_ (potato peel) exhibited significant positive effect on amylase production as shown in quadratic equation. The result clearly indicated that the amylase production increased as the concentration of potato peel increased. The interactive effect of potato peel (X_2_) and rape seed cake (X_3_) also positively affected the amylase production whereas the interactive effect of feather meal and potato peel (X_1_X_2_) negatively affected the production of amylase. This means that the increase in concentration of feather meal with potato peel would reduce the production of amylase. The 3D response surface plot has been shown in Fig. 1D–F.

### 3.5 Biosurfactant production

The value of emulsification index of biosurfactant production varied from 12.3 to 38.6% (Table 1). The *P*-value for the model (0.005) suggested the significance of the model and the value of “Lack of fit” (0.1) indicated that the obtained experimental data was good fit with the model (Table 2). The response of biosurfactant production (Y_3_) regression by strain PF1 can be expressed in terms of following regression equation:

$${\text{Y}}_{3}=23{\text{.70+1.21X}}_{1}-{\text{0.74X}}_{2}{\text{+8.67X}}_{3}{\text{+1.13X}}_{1}{\text{X}}_{2}-{\text{1.13X}}_{1}{\text{X}}_{3}-{\text{0.19X}}_{2}{\text{X}}_{3}-\text{0.36}{\text{X}}_{1}^{2}\text{+0.26}{\text{X}}_{2}^{2}\text{+0.44}{\text{X}}_{3}^{2}$$

The value of correlation coefficient (R^2^) for biosurfactant production was found to be 0.83 indicated a good fit. The quadratic equation showed that the variable X_3_ (rape seed cake) significantly increased biosurfactant production. While the variable X_2_ exhibited negative effect on biosurfactant production. The interaction of variable X1 and X2 also positively influenced the biosurfactant production. However the effect of X_2_ and X_3_ negatively influenced the biosurfactant production. The 3D response surface plot has been shown in Fig. 1G–I.

### 3.6 Validation of the model

**Fig. 2:**
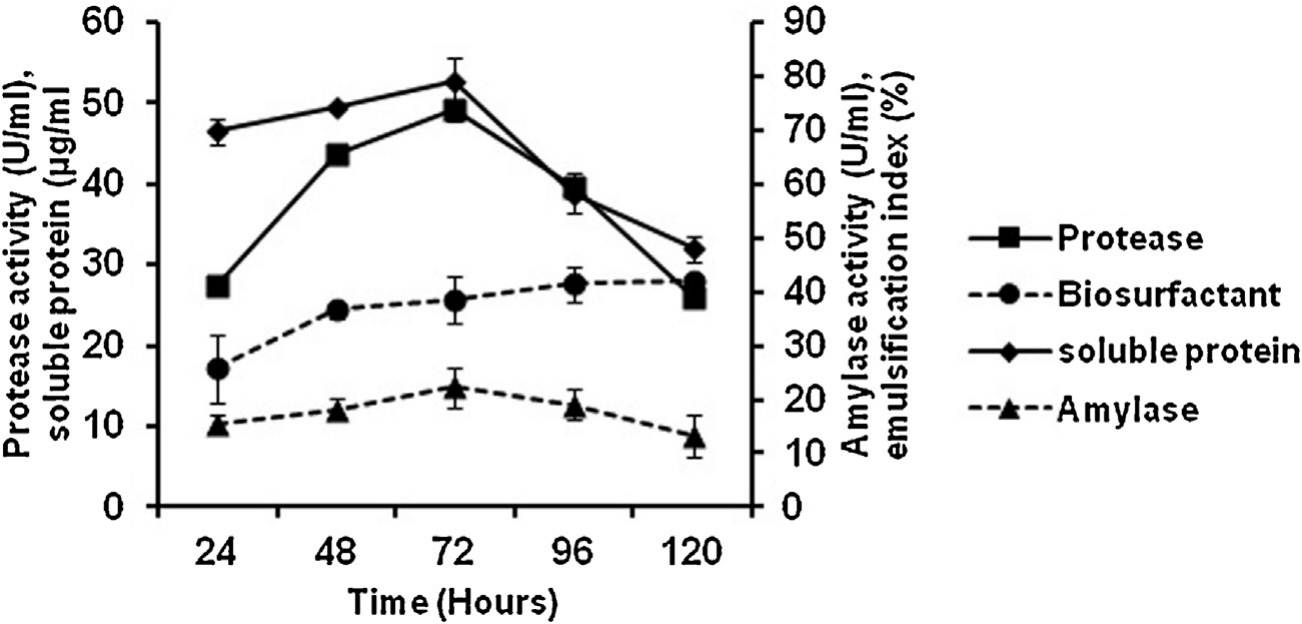
Profile of protease, amylase, soluble protein and amino acid production by *Bacillus subtilis* PF1 in the optimized media. The result is represented as mean ± standard error of 3 independent variables.

To validate the model, time course study of protease, amylase and biosurfactant production by strain of *Bacillus subtilis* PF1 was evaluated in presence of culture optimized media. The result is depicted in Fig. 2. The production of protease in un-optimized medium was 21.49 U/ml as compared to 49.14 U/ml protease in optimized media. This result confirmed that a 2.28 fold increase in protease production was observed in optimized medium. Similarly, 0.84 fold increase in amylase production and 1.2 fold increase in biosurfactant production was observed in optimized media (16.39 U/ml amylase and 15% biosurfactant in the un-optimized medium as compared to 19.13 U/ml amylase and 17% biosurfactant in the optimized medium).

### 3.7 Partial Purification of protease and PAGE

**Fig. 3:**
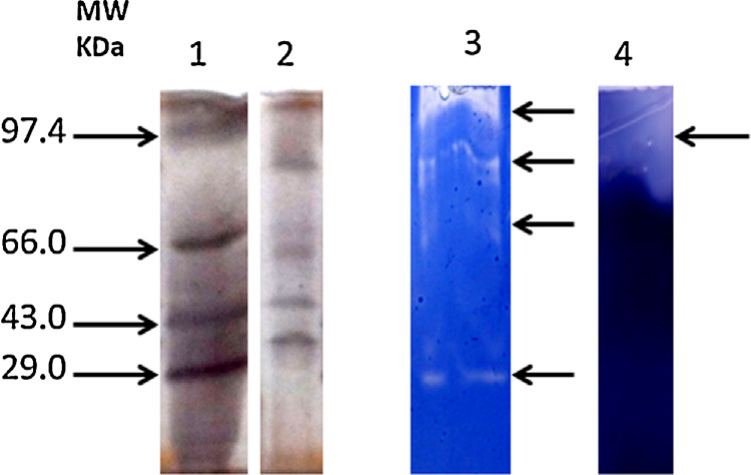
SDS–PAGE (12%) and zymography analysis of the protease. Lane 1: protein. Markers from 97 kDa to 29.0 kDa: lane 2. Partially purified protease and amylase obtained after ammonium sulphate precipitation: lane 3. Activity staining of partially purified protease: lane 4. Activity staining of partially purified amylasese obtained after ammonium sulphate precipitation.

The crude enzyme present in supernatant of culture was subjected to ammonium sulphate precipitation. The precipitate after dialysis resulted in enzyme preparation with approximately 1.91 fold purification (data not shown). The partially purified enzyme was subjected to native PAGE zymography to determine the molecular mass and number of amylases and proteases. Fig. 3 indicated the *Bacillus subtilis* PF1 produces at least four proteases ranges from approximately 97.4 to 45 kDa (lane 3) and a single amylase (lane 4) in optimized medium. The corresponding five bands could be observed in SDS PAGE gel (lane 2).

### 3.8 Effect of pH and temperature and stability on activity of protease and amylase

**Fig. 4:**
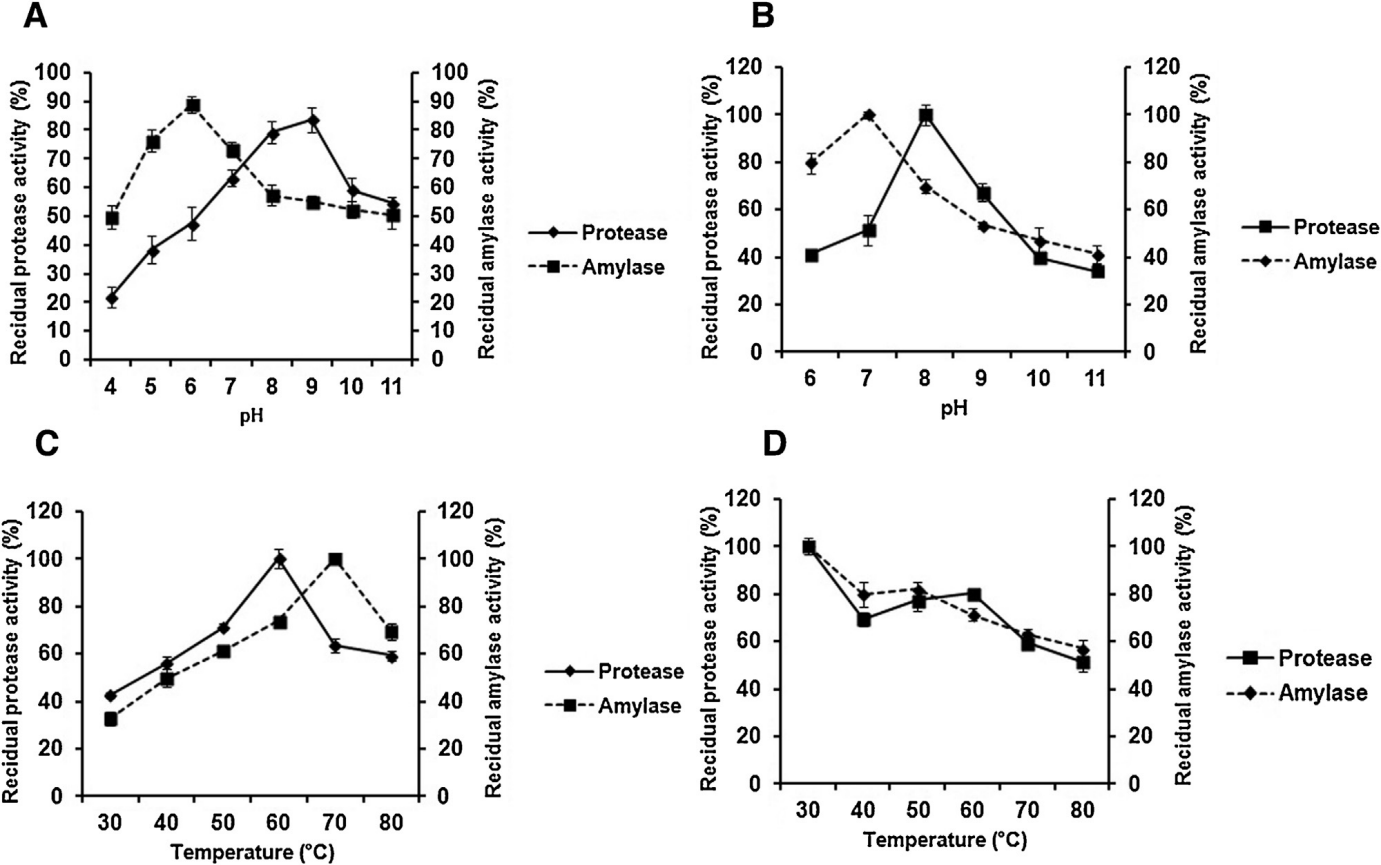
(A) Effect of pH on activity of partially purified protease and amylase. (B) Effect of pH on stability of partially purified protease and amylase. (C) Effect of temperature on partially purified protease and amylase activity. (D) Effect of temperature on partially purified protease and amylase stability. The result is represented as mean ± standard error of 3 independent variables.

The effect of different pH on enzyme activity of protease and amylase has been shown in Fig. 4A. The effect of pH on proteolytic and amylolytic activities of partially purified enzyme was determined over a pH range from 4.0 to 11.0. The protease and amylase was highly active in the pH range of 6.0–10.0. An optimum pH 9.0 was recorded for protease and pH 6.0 for amylase activity (Fig. 4A). The stability of partially purified protease and amylase was also estimated after 3 h incubation at pH 6.0–11.0. Fig. 4B displays the stability profile of protease and amylase. It was observed that both the protease and amylase exhibited stability at pH 6.0–10.0. Protease showed reduced activity to some extent after pH 9 and amylase after pH 10.0. Protease and amylase retained their 39.1 and 41.2% residual activity respectively at pH 11.0. The temperature activity profiles for both enzymes are shown in Fig. 4C. Partially purified protease was active at temperatures ranging from 50 to 80 °C and exhibited optimum activity at 60 °C. Furthermore, the protease exhibited 63.43 and 59.21% relative activity at temperature 70 and 80 °C respectively. The amylase was active at temperatures ranging from 60 to 80 °C and had optimum activity at 70 °C. The relative activity of amylase at 80 °C was 69.3%. Fig. 4D shows the effect of temperature on enzyme stability. Protease and amylase presented stability at temperature ranging from 30 to 60 °C after 3 h of incubation. Protease retained 63.2 and 56.9% relative activity at temperature 70 °C and 80 °C respectively. Similarly amylase exhibited 59.3 and 51.4% relative activity at temperature 70 °C and 80 °C.

### 3.9 Effect of metal ions, organic solvents, and surfactants

Effect of metal ions and chemicals on protease activity is depicted in Table 3. Among the metal ion examined for the protease and amylase activity, Ca^2+^ and Mg^2+^ ions exhibited a stimulatory effect. Ca^2+^ ion enhanced 97% protease activity and 71% amylase activity. Similarly, Mg^2+^ enhanced 22% protease activity and 71% amylase activity. Other metal ions namely Na^+^, Zn^2+^, and Co^2+^ did not display any considerable effect on enzyme activity. However, protease activity was inhibited significantly in presence of Hg^2+^ (91.7%) and amylase activity was found to be inhibited by Cu^2+^ (80.89%). The effect of different chemicals, detergents and protease inhibitors are shown in Table 3. About 87% increase in protease activity and 30% increased amylase activity was observed in addition of 1% DMSO as compared to control. Protease and amylase were found to be stable in presence of 10 mM SDS and exhibited 73.3% residual protease activity and 62.91% residual amylase activity under standard assay conditions. A moderate activity was recorded in presence of 2-mercaptoethanol and glycerol. The protease activity was strongly inhibited in presence of 10 mM PMSF (97%). EDTA negatively affects the enzyme activity of both protease and amylase and retained only 16.8% keratinolytic and 19.37% amylolytic activity at 10 mM concentration.

### 3.10 Compatibility of amylase and protease with commercial detergents

**Fig. 5:**
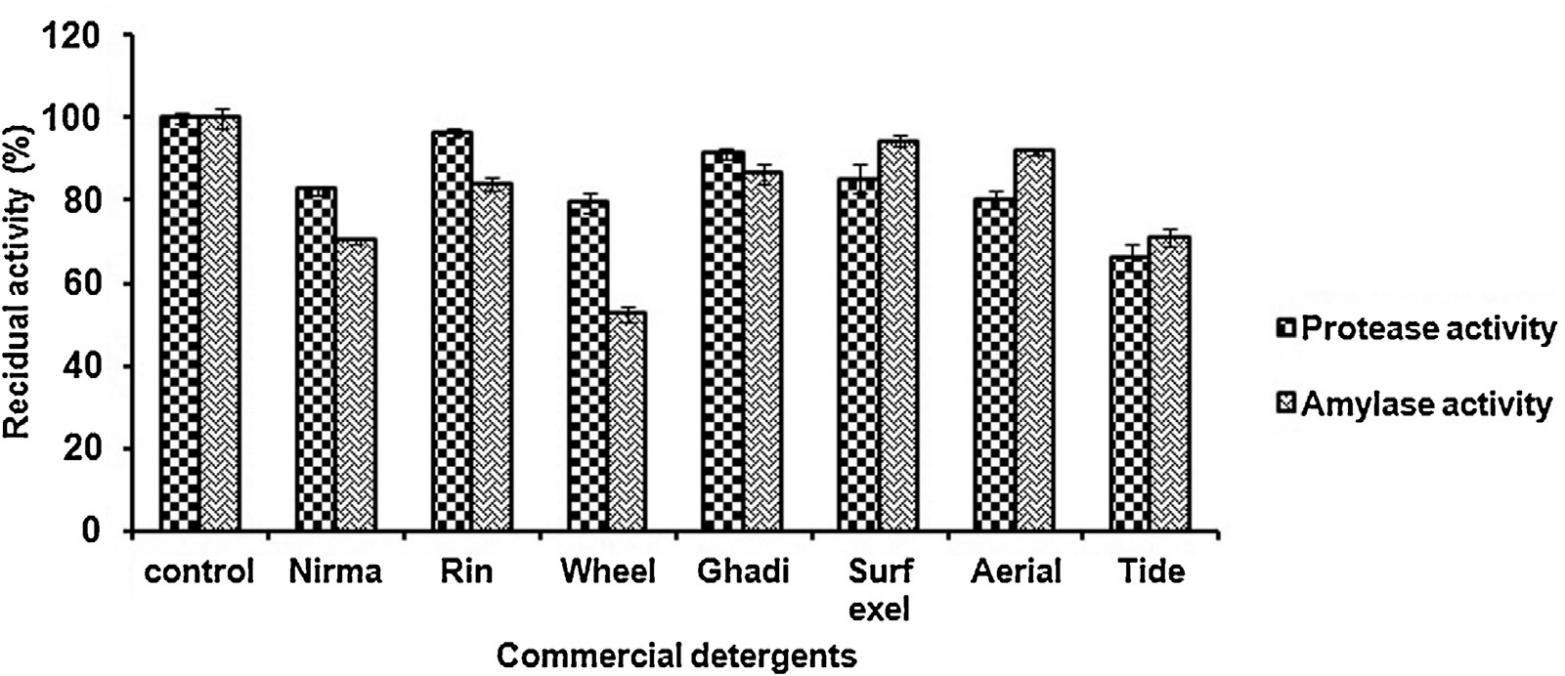
Compatibility of partially purified protease and amylase with commercially available detergents. Protease and amylase activity in the absence of detergent was considered as 100% activity and other values were compared with that. The result is represented as mean ± standard error of 3 independent variables.

In order to check the compatibility of protease and amylase with commercial solid detergents, partially purified enzyme preparation was pre-incubated in the presence of various deactivated commercial laundry detergents for 1 h at 50 °C. The solid detergents were diluted in tap water to a final concentration of 7 mg/ml to simulate washing conditions. The data presented in Fig. 5 indicated that the protease and amylase mixture was extremely stable towards all solid detergents. The protease retaining 96.4% initial activity in the presence of Rin and more than 80% and with Surf excel and Arial after 1 h incubation. Likewise, more than 90% amylase activity was recorded in presence of Arial and Surf excel.

### 3.11 Effect of pH and temperature and pH on biosurfactant

**Fig. 6:**
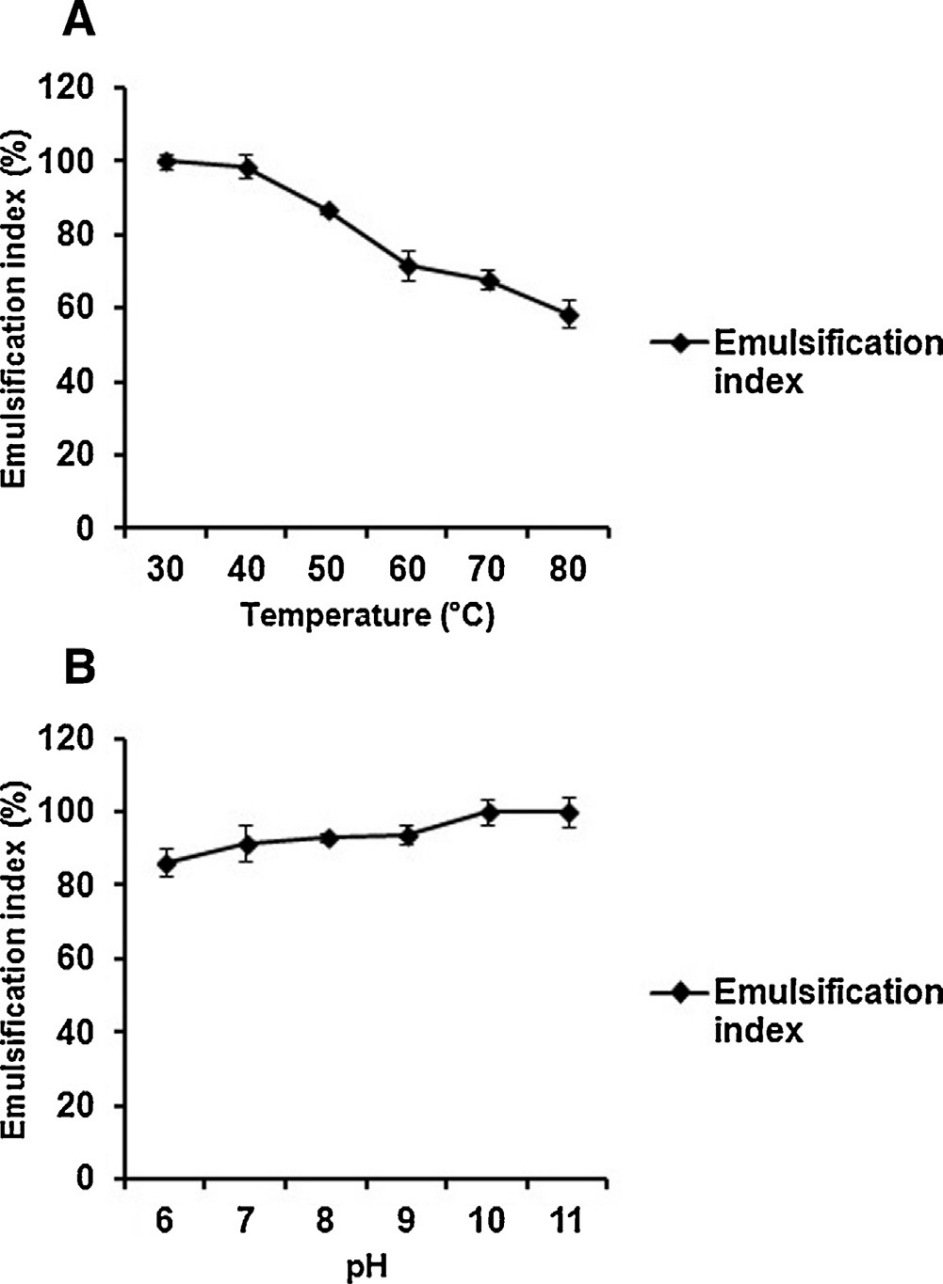
(A) Effect of temperature and (B) pH on biosurfactant activity.

The biosurfactant exhibited maximum stability at pH 6.0–11.0 displayed 100% activity (in terms of emulsification index) at pH 10.0 and 11.0 (Fig. 6A). The maximum stability was recorded between temperature ranges from 30 to 60 °C and 100% activity was observed at 30 °C temperature (Fig. 6B).

### 3.12 Washing performance of protease, amylase and biosurfactants

**Fig. 7:**
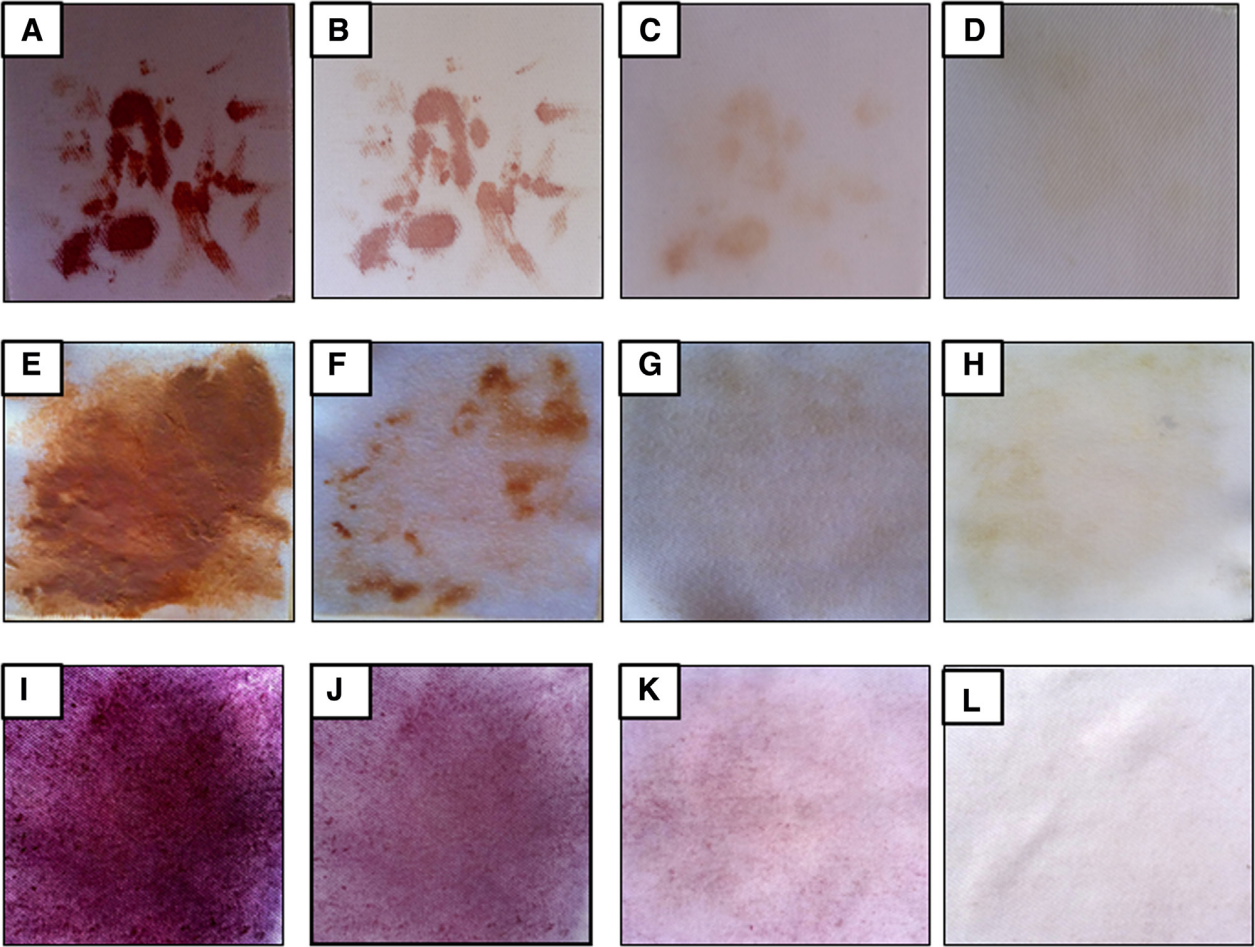
Washing performance of protease, amylase and biosurfactant from *Bacillus subtilis* PF1 (A) cloth stained with blood; (B) blood-stained cloth washed with water only; (C) blood-stained cloth washed with SDS only (D) blood-stained cloth washed with SDS added with protease, amylase and biosurfactant; (E) cloth stained with chocholate stain; (F) chocholate stained cloth washed with water only; (G) chocolate-stained cloth washed with SDS only (H) chocolate-stained cloth washed with SDS added with enzyme extract and biosurfactant; (I) cloth stained with beet root juice (J) beet root-stained cloth washed with water only (K) beet root juice stained cloth washed with SDS only (L) beet root juice stained cloth washed with SDS added with enzyme extract and biosurfactant.

Cotton cloths washed in three different sets of washing solutions (as detailed in Section 2.16) indicated that a combination of crude extract of protease, amylase and biosurfactant preparation and SDS together perform best washing (Fig. 7A–I). Although blood stain (protein rich), chocolate (greasy) and beet root juice spots (starch rich) were observed to be fainted using SDS alone; but addition of crude extract of protease, amylase and biosurfactant (with 49.14 U/ml protease, 19.13 U/ml amylase activity and 17% emulsification index) enhanced the stain removal efficiency. This result indicated that the co-produced protease, amylase and biosurfactant could be employed as potential detergent additive.

## 4 Discussion

The present report describes the multifunctional characteristic of keratinolytic bacterial strain *Bacillus subtilis* PF1. Strain PF1 could produce keratinolytic protease, amylase and biosurfactant simultaneously in the single medium containing cost effective agroindustrial wastes namely feather meal, potato peel and rape seed cake. Most of the researches have demonstrated that the mixture of protease and amylase can be used in detergent additives using glucose or starch, together with expensive nitrogen sources such as gelatine, yeast extract, or casein [20], [21], [22], [23]. However, only few studies have emphasised on protease and amylase production using inexpensive carbon and nitrogen sources such as soybean meal, wheat flour [19], [24] and tuna fish powder, red koji and sal deoiled cake [8], [25], [26]. Poultry feathers are ubiquitous and an environmental pollutant; therefore they can be utilized as a low cost substrate for the production of enzymes [27]. In addition, potato peel is the good source of starch and generated as by-product from potato chips industries.

The response surface methodology is an excellent approach to improve the industrial production of enzymes as it helps to optimize the media in relatively less time and with relatively less effort as compared to conventional one-variable-at-a time approach. Therefore, co-production of protease, amylase and biosurfactant was optimized using RSM approach. Fig. 1 shows the response for the interactive factors feather meal, potato peel and rape seed cake when the inoculum density, incubation time and phosphate levels were fixed at their optimum values 1% (v/v), 48 h and 0.07% (w/v), respectively. The results indicated that the production of these three metabolites was enhanced by increasing the concentration of feather meal, potato peel and rape seed cake in the medium up to a certain extent. An increase in the concentration of rape seed cake up to 6 g/l showed an increase in protease and amylase production however above this concentration a decrease in production was observed. However, an increase in the concentration of feather meal and potato peel did not reduce the biosurfactant production, which can be seen in 3D plot which is flattened with more and more points moving towards higher emulsification index. Co-production of protease and α-amylase from *Bacillus licheniformis* NH1 have been earlier reported by Hamidet et al. [2]. For instance concomitant production and downstream processing of alkaline protease and biosurfactant from *Bacillus licheniformis* RG1 have also been reported by Ramnani et al. [13]. To best of our knowledge this is the first report describing the simultaneous production of protease, amylase and biosurfactant in a single media. Before starting the experiments it was kept in consideration that the feather meal would provide nitrogen source for amylase production along with potato peel starch. The potato peel starch would slowly release carbon source in media and complement feather meal to produce protease and the rape seed cake would promote the production of biosurfactant.

Production of multiple proteolytic enzymes and a single high molecular weight amylase by *Bacillus subtilis* PF1 has been also demonstrated in co-produced protease and amylase from *Bacillus licheniformis* NH1 [2]. This indicated that the protease and amylase of *Bacillus subtilis* PF1 and *Bacillus licheniformis* are almost similar. The activity staining of amylase confers the compatibility of amylase with protease when they were simultaneously produced. Presence of multiple proteases is advantageous for industry as they can enhance the industrial processes by working in a mutualistic manner.

Biochemical characterization of any enzyme is extremely important before addressing its biotechnological potential or industrial applications [28]. The biochemical characterization of partially purified protease exhibited appropriate activity in neutral to alkaline pH near 7.0–9.0 and had optimum pH 9.0. More than 50% protease activity was lost after pH 9.0. Our report is in accordance with detergent stable serine protease from *Bacillus circulans* which reveal activity over alkaline range of pH [29]. The amylase was found to be active in pH range of 6.0–8.0, with optimum activity at pH 6.0. For instance, activity of amylase at this range was reported by Hmidet et al. [2]. This is also in agreement with the report of Asgher et al. [29]. The protease and amylase was demonstrated to be stable at neutral to alkaline pH (6.0–9.0) after 3 h of incubation. This indicated that the simultaneously produced protease and amylase are good candidate for detergent ingredients since detergents solution’s pH is generally between 9.0 and 10.5. The optimum activity at alkaline pH is an important factor required in almost all detergent-enzymes [30].

The activity of partially purified protease and amylase exhibited optimum activity at 60 °C and 70 °C respectively. Most of the protease and amylases reported so far were maximally active at 50–70 °C such as protease from *Bacillus licheniformis* ER-15 exhibited optimum activity at 60 °C [4]. For instance, optimal activity of protease from *Bacillus cereus* LAU 08 was determined at 50 °C [31]. Similarly, amylases from *Aspergillus flavus* TF-8 and from *Aspergillus oryzae* S2 was optimally active at 60 °C [8], [26]. The co-produced protease and amylase were found to be thermostable as both enzymes retained about 60% residual activity at 70 °C and near about 50% activity at 80 °C after 3 h of incubation. These findings are in accordance with several earlier reports presenting the thermostability of protease and amylase respectively [3], [32], [33].

Along with activity and stability in presence of high pH and temperature the enzymes used as detergent additives must be compatible with commonly used detergent components such as surfactants, bleaches, oxidizing agents and other additives which might be present in the detergent formulation [34]. The effect of various cations on enzyme activity was investigated at 2 mM concentration. The increment in enzyme activity of both co-produced protease and amylase in presence of Ca^2+^ and Mg^2+^ ion suggest the role of these ions in enzyme stimulation possibly by acting as salt or ion bridges that stabilize the enzyme in its active conformation and might protect the enzyme against thermal denaturation. Prakash et al. and Correa et al. [33], [35] have also reported the stimulation of amylolytic activity in presence of Ca^2+^. The enhancement of keratinolytic activity in presence of Mg^2+^ ions has also been demonstrated in keratinolytic serine alkaline proteinase from *Streptomyces* sp. strain AB1 [3]. The protease activity was strongly inhibited by Hg^2+^ (91.7%). This indicated the loss of protease activity in presence of Hg^2+^ is due to inactivation. The inhibition by Hg^2+^ is also in agreement with the fact that Hg^2+^ inactivates the enzyme activity by oxidizing the thiol group of cysteine and can react with tryptophan residues and carboxyl group of amino acid present in keratinase/protease [36].

The effect of various organic solvents at their different concentrations on protease and amylase stability reveal that the co-produced protease and amylase were highly stable and exhibited enhanced activity in presence of Triton X-100 and H_2_O_2._ Further, they exhibited slightly reduced activity against the strong anionic surfactants particularly SDS. The activity of both enzymes was found to be stable in presence of DMSO. These characteristics of co-produced protease and amylase facilitate their incorporation as detergent additives because surfactant and oxidizing agents are present in detergent formulations. Few studies have reported the oxidant and surfactant stable activity of wild type alkaline protease and amylase [21], [36].

Inhibition of protease activity in presence of PMSF suggested that the co-produced protease is a member of serine-protease family of enzymes. Most of the proteases reported so far belong to the serine protease family [17], [35]. Furthermore, slight reduction of enzyme activity in presence of EDTA indicated that the enzymes are metallo-type or metal ions are required for enzyme activity or stability. The amylase and protease activity was also considerably reduced in presence of β-mercaptoethanol suggesting that the co-produced protease and amylase are not thiol activating enzymes. However, protease retained 36.71% activity in presence of 0.1% and 28.13% activity in presence of 0.5% beta mercaptoethanol as compared to control. This result indicated that the stability of protease in presence of β-mercaptoethanol. This report is similar to various reports [9], [37].

The produced biosurfactant was stable in the presence of alkaline condition and moderate to high temperature which is required for good detergent additive. The presence of biosurfactant with enzyme preparation can help in better removal the stains especially greasy stains by reducing the surface tension of water [13]. It would also reduce the use of synthetic detergents which are generally not easily degradable in the environment. The role of biosurfactant in detergent action is well established. For instance, Sajna et al. [10] have reported the potential application of biosurfactant from *Pseudozyma* sp. NII 08165 as laundry detergent additive. Role of sophorolipids, yeast glycolipid biosurfactants, as biodegradable low-foaming surfactants have also been reported by Hirata et al. [11]. Furthermore, the crude cyclic lipopeptide (CLP) biosurfactants from *Bacillus subtilis* and rhamnolipids have been demonstrated as laundry detergents additives [38].

Ionic surfactants, bleaching agents and water softner builder etc. are the essential parts of commercial detergents therefore the compatibility of enzymes with this component is indispensable. However, the amylase was more sensitive in presence of some detergent and lost its activity more as compared to protease. This might be due to the activity of protease or due to the inhibitory activity of detergent components. This result is in close agreement with those reported by Roy and Mukherjee [28].

The mixture of protease, amylase and biosurfactant resulted in better stain removal from cotton fabrics as compared to that of SDS and water alone. Literature survey suggested that the amylase can also successfully remove starch from cotton fabrics, thereby: preventing the warp thread from breaking off during the weaving process [28]. This reinforces the potential application of protease, amylase and biosurfactant preparation as additives to laundry detergent formulations and for desizing activity in textile industries. The washing performance of alkaline protease and amylase were reported separately in most of the references [28], [36]. In few reports the mixture of protease and amylase were used [2]. We believe that the mixture of protease, amylase and biosurfactant would remove stains more efficiently.

## 5 Conclusion

An overall 2.28% increase in protease, 0.85% increase in amylase production and 1.2% increase in biosurfactant production was achieved with optimized media. In this report we present a cost effective method to produce enzyme and biosurfactants required for efficient washing. We feel that it will reduce overall expenditure on detergent ingredients. This will not only protect environment from accumulation of feathers but also economically utilize the agro industrial wastes such as potato peel and rape seed cake for production of useful enzymes. The simultaneously produced protease and amylase were not only stable but also exhibited increased activity in presence of surfactants and oxidizing agents that make these enzymes suitable as detergent additive. The stability of biosurfactants in broad range of temperature and alkaline environment suggested its potential applications in laundry detergents.

## Figures and Tables

**Table 1:** Central composite design of the study using three independent variables. (A) The results showing observed and predicted values.

Run	Variables			Protease activity (U/ml)		Amylase production (U/ml)		Biosurfactant production Emulsification index	
	X_1_	X_2_	X_3_	observed	predicted	observed	predicted	observed	predicted
	FM (g/l)	PP (g/l)	RC (g/l)						
1	12.5	12.5	6	50.1	48.85	15.25	16.56	22.1	23.70
2	12.5	12.5	6	59.3	59.30	16.67	16.56	24.28	23.70
3	0.113	12.5	6	14.18	8.84	11.17	14.53	12.9	19.33
4	20	5	10	44.79	49.44	4.22	7.40	29.7	33.22
5	20	20	10	36.5	33.36	21.48	19.18	32.17	33.29
6	5	20	2	26.25	22.05	27.39	22.40	15.5	11.99
7	12.5	12.5	12.7	53.65	46.83	16.96	13.52	38.6	39.52
8	12.5	12.5	0.727	24.71	30.88	18.68	19.60	11.3	10.36
9	12.5	12.5	6	54.19	48.85	15.4	16.56	27.8	23.70
10	20	20	2	31.6	32.75	25.01	24.20	14.1	17.05
11	5	5	2	11.1	14.73	6.08	6.57	16.12	15.01
12	12.5	12.55	6	39.15	48.85	18.91	16.56	26.1	23.70
13	5	5	10	33.74	33.09	5.36	4.36	36.4	33.46
14	12.5	0.113	6	40.8	42.61	4.61	4.96	27.3	25.68
15	12.5	12.5	6	37.4	48.85	15.8	16.56	18.6	23.70
16	5	20	10	18.22	29.82	27.9	28.07	32.24	29.00
17	12.5	25.11	6	37.91	35.24	22.7	28.17	21.6	23.20
18	25.11	12.5	6	26.37	31.59	17.28	18.60	29.83	23.39
19	20	5.0	2	48.03	38.25	22.28	20.31	12.3	15.55
20	12.5	12.5	6	52.41	48.85	18.12	16.56	23.3	23.70

FM: feather meal; PP: potato peel; RC: rape seed cake.

**Table 2:** Results of ANOVA analysis.

	Protease	Amylase	Biosurfactant production
P value	0.02	<0.0001	0.005
Lack of fit	0.4	0.3	0.12
Mean	37.13	16.56	23.61
CV	24.48	17.83	19.27
R^2^	0.8	0.88	0.83
Adequate precision	6.3	13.62	9.06

**Table 3:** Effect of metal ions, chemicals, detergent and enzyme inhibitors on enzyme activity. Protease activity measured in the absence of any metal ions, chemicals, detergent and enzyme inhibitors was taken as control (100%). The non treated enzyme added with 1 mM EDTA was considered as 100% for metal ion assay. Residual enzyme activity was measured under standard assay conditions.

Bivalent ions/enzyme inhibitors/other chemicals	Concentration	Residual activity of protease%	Residual activity of amylase%
Control	–	100	100
Na^+^(NaCl)	2 mM	66.71 ± 1.38	58.96 ± 3.56
Zn^2+^(ZnCl_2_)	2 mM	42.17 ± 1.21	20.8 ± 0.91
Co^2+^(CoCl_2_)	2 mM	51.18 ± 2.33	55.18 ± 1.86
Hg^2+^(HgCl_2_)	2 mM	8.3 ± 3.41	38.14 ± 2.5
Cu^2+^ (CuSO_4_)	2 mM	44.27 ± 1.29	19.11 ± 1.66
Mg^2+^(MgCl_2_)	2 mM	122.7 ± 3.21	147.31 ± 4.97
Ca^2+^ (CaCl_2_)	2 mM	197.12 ± 3.24	171.48 ± 1.67
DMSO	0.5% (v/v)	137.6 ± 2.37	117.72 ± 0.88
	1% (v/v)	181.68 ± 1.47	130.38 ± 0.45
Triton X-100	0.10%	113.06 ± 5.9	141.01 ± 4.9
	0.50%	196.29 ± 6.11	163.15 ± 6.1
H_2_O_2_	0.50%	98.87 ± 6.42	81.07 ± 4.39
	1%	73.22 ± 2.57	69.46 ± 2.09
Glycerol	0.5% (v/v)	72.17 ± 1.13	51.74 ± 5.81
	1% (v/v)	82.21 ± 3.25	58.91 ± 1.84
β-mercaptoethanol	0.1% (v/v)	36.71 ± 3.19	16.88 ± 3.62
	0.5%(v/v)	28.13 ± 2.59	9.25 ± 4.98
SDS	5 mM	86.1 ± 1.31	74.90 ± 2.75
	10 mM	73.3 ± 1.88	62.91 ± 1.9
EDTA	5 mM	29.4 ± 1.6	23.91 ± 3.34
	10 mM	16.8 ± 1.96	19.37 ± 2.7
1,10-Phenanthroline	5 mM	44.17 ± 3.15	67.29 ± 0.37
	10 mM	31.15 ± 0.71	64.86 ± 0.8
PMSF	5 mM	9.72 ± 3.08	89.12 ± 1.43
	10 mM	3.09 ± 2.23	82.73 ± 2.1

The partially purified enzyme preparation was incubated with different detergents components for 1 h at 30 °C and the remaining activity was measured under standard assay conditions.
